# Characterization of Changes in P-Wave VCG Loops Following Pulmonary-Vein Isolation

**DOI:** 10.3390/s21051923

**Published:** 2021-03-09

**Authors:** Nuria Ortigosa, Óscar Cano, Frida Sandberg

**Affiliations:** 1 I.U. Matemática Pura y Aplicada, Universitat Politècnica de València, Camino de Vera s/n, Edif. 8E, Acceso F, 46022 Valencia, Spain; 2 Servicio de Cardiología, Hospital Universitari i Politècnic La Fe, Planta 4-Torre F, Av. Fernando Abril Martorell 106, 46026 Valencia, Spain; cano_osc@gva.es; 3 Centro de Investigaciones Biomédicas en Red en Enfermedades Cardiovasculares (CIBERCV), 3, 28029 Madrid, Spain; 4 Department of Biomedical Engineering, Lund University, Box 118, 221 00 Lund, Sweden; frida.sandberg@bme.lth.se

**Keywords:** vectorcardiogram (VCG), atrial fibrillation, P wave, pulmonary-vein isolation

## Abstract

Atrial fibrillation is the most common type of cardiac arrhythmia in clinical practice. Currently, catheter ablation for pulmonary-vein isolation is a well-established treatment for maintaining sinus rhythm when antiarrhythmic drugs do not succeed. Unfortunately, arrhythmia recurrence after catheter ablation remains common, with estimated rates of up to 45%. A better understanding of factors leading to atrial-fibrillation recurrence is needed. Hence, the aim of this study is to characterize changes in the atrial propagation pattern following pulmonary-vein isolation, and investigate the relation between such characteristics and atrial-fibrillation recurrence. Fifty patients with paroxysmal atrial fibrillation who had undergone catheter ablation were included in this study. Time-segment and vectorcardiogram-loop-morphology analyses were applied to characterize P waves extracted from 1 min long 12-lead electrocardiogram segments before and after the procedure, respectively. Results showed that P-wave vectorcardiogram loops were significantly less round and more planar, P waves and PR intervals were significantly shorter, and heart rate was significantly higher after the procedure. Differences were larger for patients who did not have arrhythmia recurrences at 2 years of follow-up; for these patients, the pre- and postprocedure P waves could be identified with 84% accuracy.

## 1. Introduction

Atrial fibrillation (AF) is supraventricular tachyarrhythmia with ineffective atrial conduction due to uncoordinated atrial electrical activation. Its symptoms include palpitations, dyspnea, fatigue, and chest pain. AF is the most common type of sustained cardiac arrhythmia, and estimated prevalence is between 2% and 4% in adults, with a lifetime risk of 1 in 3 in the European Union [1]. AF is traditionally stratified into five types on the basis of the presentation, duration, and spontaneous termination of arrhythmia episodes: first diagnosed, paroxysmal, persistent, long-standing persistent, and permanent AF. Catheter ablation for pulmonary-vein isolation (PVI) is the recommended treatment to permanently restore the sinus rhythm when antiarrhythmic drugs fail [2]. Success rates for PVI are up to 80%, specially for patients with paroxysmal AF for whom recurrence rates are significantly lower after the procedure [3].

In order to personalize treatment, it is desirable to predict PVI outcome. Previous studies showed that patient characteristics such as large left atrial diameters [4] can predict time to recurrence, which is a major determinant of outcome [5,6]. Other studies focused on analyzing atrial electrical activity in the electrocardiogram (ECG). Such analysis can provide information on the atrial substrate and may be used to identify the spontaneous reconnection of previously isolated pulmonary veins after catheter ablation, which can predict arrhythmia recurrence [7]. A decrease in F-wave amplitude was shown to be associated with AF recurrence after ablation [8]. Some studies focused on the spectral content of F waves, of which the dominant frequency was found to significantly decrease during catheter ablation [9], and their related spectral features are useful in predicting the procedure outcome [10]. Further, a decrease in P-wave area is associated with AF recurrence [11,12,13].

Nonetheless, it is P-wave duration that was indicated by most references as the clinical feature that is able to predict AF recurrence after pulmonary-vein isolation [14,15,16,17]. Shortened P-wave duration is associated with a successful PVI outcome and lower rates of AF recurrence. However, despite the growing knowledge in the field, the identification of changes induced in the atrial substrate that yield a successful PVI are of paramount importance for the clinical management of the arrhythmia [18,19].

In this work, we characterize atrial activity (focusing on P waves) before and after PVI by noninvasive ways, and provide analytical evidence that this procedure modifies the atrial substrate [20]. We analyze spatial ECG information manifested as vectorcardiographic loops of P waves of patients with paroxysmal AF. Thus, morphological changes and time measurements related to atrial activity are described to reveal how these changes are associated with successful PVI outcome and AF recurrence [21].

The purpose of the present study is to analyze changes in P-wave loop morphology following PVI, and investigate the relation between such changes and AF recurrence. Further, we investigate if combining P-wave loop morphology with other ECG-derived characteristics improves characterization.

## 2. Materials and Methods

### 2.1. Dataset

Fifty subjects with paroxysmal AF who had undergone PVI at the arrhythmia unit of the Hospital Universitari i Politècnic La Fe in Valencia, Spain were included in this study. A standard 12-lead ECG was acquired at 1000 Hz sampling frequency throughout the procedure using a Labsystem Pro EP Recording System. ECG recording continued for at least 30 min after successfully finishing catheter ablation. Recurrence information was obtained at a 2 year follow-up: 39 subjects maintained sinus rhythm and 11 subjects suffered AF recurrences. Details on the population cohort are shown in Table 1.

For the present study, one 60 s segment before the procedure and one 60 s segment after the procedure were extracted from ECG recordings for analysis. The segments were selected as close as possible to the beginning and end of the recording, respectively, provided that the patients were in sinus rhythm and that signal quality was sufficient.

### 2.2. Signal Preprocessing

First, the ECG was filtered in order to remove power-line interference and baseline wander using notch filtering at 50 Hz and cubic splines, respectively [22].

P-wave boundaries were automatically delineated using the multilead wavelet-based approach presented in [23,24]. This method enhances the delineation system based on single lead [25] by projecting the wavelet transform obtained from vectorcardiographic loops into a direction that optimizes signal/noise ratio, and so the delineation. The detected P-wave onset and offset points were manually reviewed, and in cases where automatic detection had failed, the points were manually corrected.

P waves of insufficient signal quality were identified on the basis of cross-correlation. For each recording, a P-wave template was obtained as the average of all P waves in the recording. P waves with cross-correlation below 0.9 to the corresponding P-wave template were considered to be of insufficient signal quality and excluded from further analysis.

### 2.3. P-Wave Loop Characterization

A vectorcardiogram (VCG) was synthesized from the 12-lead ECG of each P-wave using the Kors matrix [26], thus obtaining three orthogonal Frank leads (X,Y,Z). The Kors matrix was chosen since recent references [27,28] showed that better estimates for spatial angles are yielded when synthesizing VCG by Kors rather than using the inverse Dower matrix [29].

For each P wave, three eigenvalues were obtained by applying singular-value decomposition to the matrix formed by the three orthogonal VCG leads expressed in mV. These three eigenvalues are related to the dimensions of the P-wave loop in the 3D space. If we denote these eigenvalues as $\lambda_{i}$, where i=1,2,3 and $\lambda_{1}\geq \lambda_{2}\geq \lambda_{3}$, we can define two morphology parameters:

(1) $$\rho =\frac{\lambda_{2}}{\lambda_{1}}$$

(2) $$\varphi =\frac{\lambda_{3}}{\lambda_{1}+\lambda_{2}}$$

Parameter ρ quantifies the loop’s roundness, with larger values corresponding to rounder loops, and smaller values to more ellipsoidal loops. The second parameter, φ, quantifies the loop’s planarity, with smaller values for more planar loops, i.e., loops that can mostly be fitted to a plane. ρ takes values in the [0–1] range, whereas φ takes values in the range of [0–0.5].

### 2.4. Main Propagation Direction

The main direction of propagation in each recording was estimated by the dominant vector of the average P-wave loop. First, the spatial and time alignments of P-wave loops were performed by scaling, rotating, and applying time synchronization according to the method presented in [30]. These three transformations can be described by

(3) $$Z=\alpha QZ_{R}J_{\tau },$$

where α is a positive parameter that controls scaling, *Q* is a 3 × 3 matrix that controls the rotational changes of the heart, and $J_{\tau }$ models the time synchronization by time shift τ=−Δ,…,Δ. *Z* and $Z_{R}$ denote the 3 ×L and the 3 ×(L+2Δ) matrices containing in each row *L* or L+2Δ samples of the observed loop and reference loop, respectively. Reference $Z_{R}$ was defined as the loop corresponding to the P wave with the maximal amplitude in lead V1. This loop is associated to a specific point in the respiratory cycle, and we aimed to align all loops to this point (see [30] for a detailed explanation of optimal estimates for the different alignment parameters and the alignment process).

The average P-wave loop was obtained from the aligned P-wave loops, and the dominant vector was extracted using eigenvalue decomposition; the eigenvector associated with the first eigenvalue was considered to be main direction of propagation $v_{p}$. Changes in the main direction of propagation were quantified by the angle difference between $v_{p}$ before and after PVI, denoted θ.

### 2.5. Time Intervals

Furthermore, changes in P-wave duration, RR and PR intervals in response to PVI were investigated. P-wave duration was obtained using the P-wave onset and offset, determined as described in Section 2.2. The PR interval was obtained as the time between P-wave onset and QRS onset [31].The position of the R peak was determined from the detected QRS complexes using a wavelet-transform delineation technique [25]. RR interval was obtained as the time between subsequent R peaks.

### 2.6. Statistical and Cluster Analyses

Features characterizing individual P-wave loops and time intervals were averaged to obtain an estimate of the overall characteristics in each recording; averages are denoted as ${\bar{\lambda }}_{1},{\bar{\lambda }}_{2},\ldots$. Further, the intrarecording variation of the features was quantified by mean absolute deviation (MAD). For each set of data $X=\{x_{1},x_{2},\ldots x_{n}\}$, MAD was obtained as $MAD=1/n\sum_{i=1}^{n}\left|x_{i}-\mu \left(X\right)\right|$, where μ(X) is the mean of the dataset.

The recording averages and MAD of the features pre- and post-PVI are presented as gross mean ± standard deviation, and gross median and interquartile range, respectively, depending on the Shapiro–Wilk normality test. Further, a paired Student’s *t*-test or Wilcoxon–Mann–Whitney test, respectively, was applied to determine if differences were significant.

Moreover, cluster analysis was performed to investigate if P-wave loop-morphology and time-interval characteristics could be combined to better characterize differences pre- and post-PVI. K-means++ [32] with a squared Euclidean distance metric was used for this task. The final chosen centroids were obtained after averaging the results of several different randomized seeds, and the leave-one-out approach to perform classification.

Classification performance was assessed by means of different measures:

- sensitivity: proportion of pre-PVI features correctly identified as such;
- specificity: proportion of post-PVI features correctly identified as such;
- precision: proportion of pre-PVI features correctly classified with respect to the total number identified as such; and
- accuracy: proportion of correctly classified (both pre- and post-PVI) with respect to their total number.

## 3. Results

### 3.1. P-Wave Loop Characteristics

**Figure 1:**
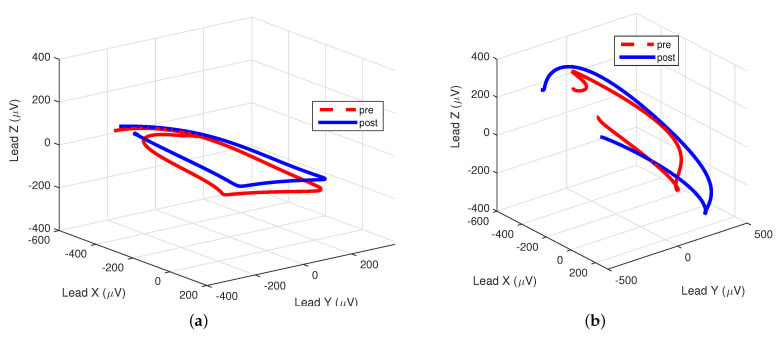
Pre- and post- pulmonary-vein isolation (PVI) loops for two different patients. (**a**) Patient with atrial-fibrillation (AF) recurrence during follow-up: ρ = 0.32 vs. 0.29, φ = 0.11 vs. 0.10, respectively. (**b**) Patient without AF recurrence during follow-up: ρ = 0.34 vs. 0.28, φ = 0.13 vs. 0.10, respectively.

Figure 1 illustrates an example of P-wave loops before and after PVI for two patients, one with AF recurrence and one without AF recurrence. After PVI, elongation of the first diagonal of the P-wave loop could be observed, corresponding to an increase in $\lambda_{1}$. Further, the P-wave loop was less round and more planar after PVI, corresponding to a decrease in ρ and φ, respectively. Changes were more prominent for the patient without AF recurrence.

The average and MAD of the P-wave loop characteristics pre- and post-PVI for all patients are summarized in Table 2. Results indicate that ${\bar{\lambda }}_{1}$ was significantly larger post-PVI, and that $\bar{\rho }$ and $\bar{\varphi }$ were significantly smaller post-PVI.

**Figure 2:**
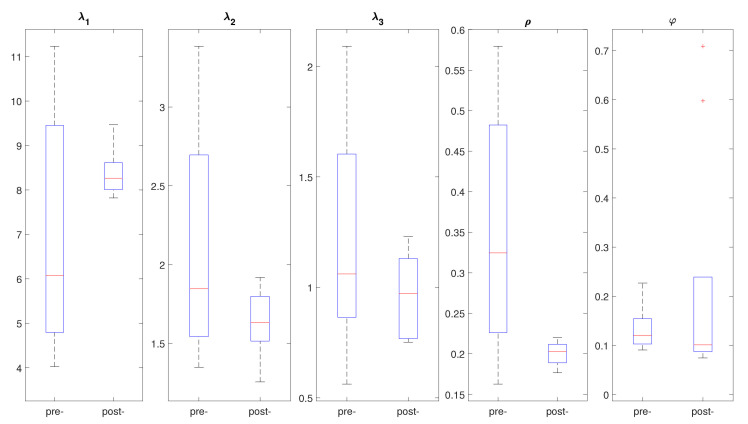
Example of box plots for different morphological features of P-wave loops under study comparing pre- and post-PVI distribution for a patient included in the study.

Results also indicate that the P-wave loops within each recording were fairly similar, as quantified by the MAD of the features. Further, the MAD of ρ was significantly lower post-PVI, indicating that P-wave loop roundness was more regular after the procedure. Figure 2 depicts an example of the box plots for the morphological loop features of a patient, comparing pre- and post-PVI features in order to illustrate within-patient variation.

**Figure 3:**
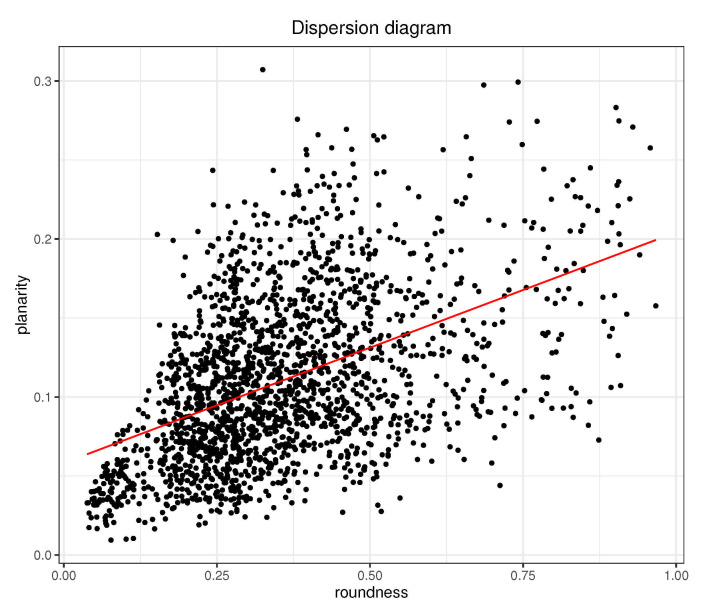
Dispersion diagram for ρ and φ for dataset including all analyzed P waves of the study. Pearson correlation is 0.47.

There was significant positive correlation between ρ and φ, as shown in Figure 3. This means that P-wave loops that were rounder were generally also less planar (since lower values for φ stand for more planar loops). No other correlations between P-wave loop characteristics were found.

### 3.2. Time Intervals

ECG-derived time measurements were also measured. Table 3 shows the average and MAD of P-wave duration, PR interval, and RR intervals for pre- and post-PVI, as well as the significance *p* value test. Results indicate that, even though all measurements were shorter after the procedure, there were no statistically significant differences unless for the RR interval, which significantly decreased.

Similar to what was found for morphological characteristics, MAD also decreased after PVI, which reflected the reduction of the dispersion associated with a more regular signal after the procedure.

### 3.3. Cluster Analysis

Table 4 individually details the classification performance for each P-wave loop morphology and time-interval feature, and for the combination of features that maximizes global accuracy. The best individual classification performance was achieved for the PR interval, followed by P-wave duration. Among P-wave characteristics, ρ provided the best classification performance.

Overall best classification performance was achieved when combining time-interval features with P-wave loop morphology features. By combining the ρ, φ, PR interval, RR interval, and P-wave duration characteristics, classification accuracy increased up to 84%, indicating that the features provide complementary information. Adding more features did not improve accuracy.

### 3.4. Differences between Patient Groups

Changes in P-wave loop characteristics, time intervals, and main direction of propagation for the group of patients with and without AF recurrence, respectively, are summarized in Table 5. In the group of patients with no AF recurrence, $\bar{\rho }$, $\bar{\varphi }$, $\bar{R}R$, and $\bar{P}$-wave duration significantly decreased, whereas ${\bar{\lambda }}_{1}$ significantly increased. For the group of patients with AF recurrence, on the other hand, ${\bar{\lambda }}_{2}$, and ${\bar{\lambda }}_{3}$ significantly decreased.

Classification performance for the group of patients without AF recurrence was 84%, while it was slightly smaller for the group with AF recurrence (81%). Classification results for individual features were remarkably better for patients who did not have AF recurrence, thus reflecting the success of the procedure.

## 4. Discussion

Several characteristics were analyzed to characterize modifications induced by catheter ablation for PVI on P-wave loops extracted from the VCG of patients with paroxysmal AF. Results showed that the first eigenvalue associated to matrices corresponding to P-wave loops significantly increased after catheter ablation. In particular, this behavior was consistent in patients for whom no AF recurrence appeared, while the first eigenvalue changed in the opposite way (decreased) for patients with recurrence. This increment of $\lambda_{1}$ is consistent with previous works that found that P-wave amplitude measured on lead V1 is larger after PVI [12], and lower in patients with AF recurrence than in those without [33].

Linked to this feature was the loop-roundness measurement (ρ), which was also notably smaller after catheter ablation and for patients without arrhythmia recurrence. Regarding intrapatient variation, Table 2 also shows that all parameters presented less mean absolute deviation after catheter ablation, despite the only feature that significantly differed being loop roundness. In this manner, changes in morphology (less round loops) could be used as the predictor of a successful procedure. Moreover, there is direct correlation between the increment of $\lambda_{1}$ and the decrement of ρ (Equation (1)). The increment of $\lambda_{1}$ is in line with obtained results in previous studies that showed an increment of P-wave amplitudes and P-wave areas after PVI in ECG recordings [12,13] and simulations [34,35].

Similar to roundness, planarity (φ) also showed a significant decrement after PVI, especially for patients without AF recurrence. Equation (2) also showed negative correlation between $\lambda_{1}$ and φ, which reflected that higher values of $\lambda_{1}$ suggest lower values of φ (i.e., more planar loops). Some works indicated that more planar loops of ventricular activity come from healthier subjects [36], and the same behavior is expected for AF patients for whom more planar loops are linked to more organized AF and decreased loop variability [21]. Presented results agree with these previous works, obtaining loops that were mostly contained in a two-dimensional plane. Furthermore, when comparing both morphology measurements (planarity and roundness in Figure 3), there was positive correlation between them, so that less round loops also seemed to be more planar.

Many recent references showed that P-wave duration decreases once catheter ablation ends [11,14,15,17,18,37], and heart rate increases (RR interval decreases) [38,39,40]. Our results are in line with these previous references. In our case, PR interval was the single feature that provided the best classification into pre- and post-PVI (see Table 4).This could be due to its connection with P-wave duration since they behave similarly: shorter P waves were observed after PVI [11], and longer PR intervals wee associated with longer P waves, with a significant predictive value of AF recurrence, and advanced left atrium remodeling due to AF [41].

We propose the combination of P-wave loop morphology measurements (roundness and planarity) and time features (RR interval, PR interval, and P-wave duration) to achieve better performance. Table 4 strengthens this premise since it shows that the combination of the five proposed features improves the characterization of pre- and post-PVI P waves.

Therefore, the presented work may help to characterize changes induced by pulmonary-vein isolation in atrial electric propagation, and how these changes are related to AF recurrence. It aimed at revealing AF recurrence by analyzing noninvasive recordings. Thus, regular checkups may allow for clinicians to manage arrhythmia progression in advance and improve AF treatment. Future work will focus on how different ablation sources may affect the different studied parameters [42,43], and will prospectively analyze how previous AF burden affects ablation outcome [3] with a larger cohort of patients.

## 5. Conclusions

The aim of the current study was to characterize P-wave loops from VCG before and after catheter ablation for pulmonary-vein isolation in paroxysmal AF patients. The use of morphology (roundness and planarity) with time measurements such as RR intervals and P-wave duration/PR intervals showed that P-wave analysis may reveal changes in the atrial propagation pattern due to the procedure, and may also help to predict AF recurrence outcome.

## Figures and Tables

**Table 1:** Baseline characteristics of study population. Hypertension defined as systolic blood pressure above 140 mmHg or diastolic blood pressure above 90 mmHg. Diabetes mellitus defined as serum fasting glucose ≥7.0 mmol/L or on medications. Hypercholesterolemia defined as cholesterol ≥6.4 mmol/L or treatment with lipid-lowering drugs. Structural heart disease defined as left ventricular hypertrophy <15 mm, left ventricular ejection fraction (LVEF) <50%, moderate or greater degrees of valvulopathy, significant coronary artery disease, or the presence of primary myocardial diseases. Detailed information about antiarrhythmic drugs is also included.

Characteristic	Number, %
Age (mean, range)	56 (26–73)
Male (n,%)	37, 74%
Hypertension (n,%)	28, 56%
Diabetes mellitus (n,%)	5, 10%
Hypercholesterolemia (n,%)	13, 26%
Left ventricular ejection fraction (mean, range)	66 (54–79)
Left atria diameter (mean, range)	38 (25–50)
Previous electrical cardioversion (n,%)	10, 20%
Time (in months) since first AF diagnosis (mean, range)	24 (11–40)
Antiarrhythmic drugs (n,%)	45, 90%
Amiodarone (n,%)	6, 12%
Flecainide/propafenone (n,%)	36, 72%
Betablockers (n,%)	30, 60%
Digoxine (n,%)	1, 2%
Nondihydropyridine calcium channel blockers (verapamil/diltiazem) (n,%)	5, 10%

**Table 2:** Pre- and post-PVI morphological features, and average mean absolute deviation (MAD) about mean for the whole study population (50 subjects). Data are mean ± standard deviation or median (interquartile range). * *p*-value < 0.05 by paired Student’s *t*-test or Wilcoxon–Mann–Whitney test.

Feature	Pre	Post	*p* Value
${\bar{\lambda }}_{1}$	5.6 (5.2–6.1)	6.0 (5.7–7.6)	0.020 *
${\bar{\lambda }}_{2}$	1.9 (1.7–2.2)	1.8 (1.7–2.1)	0.251
${\bar{\lambda }}_{3}$	0.8 ± 0.3	0.8 ± 0.3	0.280
$\bar{\rho }$	0.35 (0.32–0.39)	0.31 (0.28–0.35)	0.002 *
$\bar{\varphi }$	0.11 ± 0.04	0.09 ± 0.04	0.012 *
MAD $\lambda_{1}$	0.7 (0.5–1.5)	0.8 (0.4–1.2)	0.681
MAD $\lambda_{2}$	0.4 (0.2–0.6)	0.3 (0.2–0.5)	0.356
MAD $\lambda_{3}$	0.2 (0.1–0.2)	0.2 (0.1–0.2)	0.992
MAD ρ	0.09 ± 0.06	0.06 ± 0.05	0.030 *
MAD φ	0.03 ± 0.02	0.03 ± 0.02	0.671

**Table 3:** Pre- and post-PVI time-interval measurements, and average MAD about mean for the whole study population (50 subjects). Data are mean ± standard deviation or median (interquartile range). * *p*-value < 0.05 by paired Student’s *t*-test or Wilcoxon–Mann–Whitney test.

Feature	Pre	Post	*p* Value
$\bar{PR}$ (ms)	184 (169–205)	179 (162–210)	0.817
$\bar{RR}$ (ms)	1020 (889–1136)	934 (814–1088)	0.026 *
$\bar{P}$-wave duration (ms)	127 (123–137)	122 (121–133)	0.118
MAD PR (ms)	9 ± 19	7 ± 7	0.663
MAD RR (ms)	89 (10–50)	87 (5–32)	0.732
MAD P-wave duration (ms)	6 (3–9)	6 (3–12)	0.890

**Table 4:** Classification results when differentiating pre- and post-PVI using the different features, and the combination that maximizes global accuracy.

Feature	Sensitivity	Specificity	Precision	Accuracy
$\lambda_{1}$	0.67	0.67	0.76	0.67
$\lambda_{2}$	0.69	0.74	0.74	0.67
$\lambda_{3}$	0.64	0.64	0.66	0.63
ρ	0.66	0.73	0.68	0.69
φ	0.63	0.67	0.65	0.65
PR interval	0.81	0.78	0.81	0.79
RR interval	0.50	0.63	0.92	0.64
P-wave duration	0.72	0.74	0.77	0.72
ρ, PR, RR, φ, P-wave duration	0.84	0.85	0.89	0.84

**Table 5:** Average changes following PVI for subset of patients who had AF recurrences (11 subjects) and those who did not have AF recurrence (39 subjects) along 2 years of follow-up. * *p*-value < 0.05 by paired Student’s *t*-test or Wilcoxon–Mann–Whitney test.

Feature	AF Recurrent	No Recurrence
$\Delta {\bar{\lambda }}_{1}$	−0.5	0.8 *
$\Delta {\bar{\lambda }}_{2}$	−0.5 *	−0.1
$\Delta {\bar{\lambda }}_{3}$	−0.1 *	−0.1
$\Delta \bar{\rho }$	−0.02	−0.03 *
$\Delta \bar{\varphi }$	−0.02	−0.02 *
$\Delta \bar{PR}$ (ms)	−14	−1
$\Delta \bar{RR}$ (ms)	−83	−96 *
$\Delta \bar{P}$-wave duration (ms)	−2	−6 *
$\Delta \bar{\theta }$ (°)	20.85	34.89

## Data Availability

The data presented in this study are available on request from the corresponding author. The data are not publicly available due to ethical and privacy issues.
